# Case report: Laparoscopic approach in the treatment of presacral lipoma

**DOI:** 10.1016/j.amsu.2018.09.016

**Published:** 2018-09-21

**Authors:** Eligijus Poskus, Gabija Makunaite, Ieva Kubiliute, Donatas Danys

**Affiliations:** a Centre of Abdominal Surgery, Vilnius University Hospital Santaros Clinics, Santariskiu 2, LT-08661, Vilnius, Lithuania; b Faculty of Medicine, Vilnius University, M. K. Ciurlionio Street 21, Vilnius, LT-03101, Lithuania

**Keywords:** Retrorectal lipoma, Rare tumors, Laparoscopic surgery, Retrorectal tumor, Case report

## Abstract

Retrorectal lipoma, as well as other retrorectal tumours, is a relatively rare disorder. Retrorectal tumours accounted for 1 in 40,000 hospital admissions. We present a case of retrorectal lipoma, 15 cm × 10 cm × 8 cm in size, treated by the laparoscopic approach. The preoperative magnetic resonance imaging visualised a mass, 12 cm × 6.7 cm × 8.6 cm in diameter, in the retrorectal space, spreading toward the left obturator foramen. Surgery was indicated due to exclude malignant process certainly, because it is difficult to differentiate lipoma from low-grade liposarcoma on non invasive imaging. Laparoscopic extirpation of the tumour was performed. The overall operative time was 80 min. The diagnosis of lipoma was established on histological examination. The patient was discharged from hospital on the 2nd day after the surgery. We have found this minimally invasive operation to be an effective and well-tolerated treatment option, determined by the experience of the surgeon.

## Highlights

• Retrorectal lipoma is a benign mesenchymal tumour composed of mature adipose tissue, located in the retrorectal space [1].
• Retrorectal tumours in adults are rare [[3], [4], [5], [6], [7], [8], [9]], Jao et al. found that retrorectal tumours accounted for 1 in 40,000 hospital admissions [5].
• Miscellaneous, non classified tumours account for 10–25% of all retrorectal tumors. Retrorectal lipomas are included in this group [4,7].
• These tumours usually affect middle-aged patients and occurs twice as often in women as in men [7].
• Retrorectal lipomas, as well as other benign retrorectal tumours, are often completely asymptomatic [4,6,8,9].
• They are usually found incidentally when performing a pelvic or rectal examination [4,9].
• Retrorectal lipomas also might give nonspecific symptoms, mostly from the compression of pelvic structures, viscera and nerves. Symptoms depend on the size of the tumour, its localisation, extension [3].
• Diagnosis and treatment of retrorectal tumours, including lipomas, remain difficult. Currently, computed tomography scan used in conjunction with magnetic resonance imaging scan is the gold standard in diagnosing retrorectal tumours [4].
• These methods are used to find out the size, structure of the tumour, its relationship with surrounding organs that is a necessary information for planning surgical approach [3,4,8].
• Biopsy should not be performed before a surgery [5,6].
• All retrorectal tumours, including lipomas, should be resected [3,6,7].
• In the presenting case laparoscopic surgery was indicated due to exclude malignant process, because it is difficult to differentiate lipoma from low-grade liposarcoma on magnetic resonance imaging [10].

## 1 Introduction

Retrorectal lipoma is a benign mesenchymal tumour composed of mature adipose tissue, located in the retrorectal space [1]. Retrorectal tumours in adults are rare [[3], [4], [5], [6], [7], [8], [9]], Jao et al. found that retrorectal tumours accounted for 1 in 40,000 hospital admissions [5]. Miscellaneous, non classified tumours account for 10–25% of all retrorectal tumors. Retrorectal lipomas are included in this group [4,7]. These tumours usually affect middle-aged patients and occurs twice as often in women as in men [7]. Retrorectal lipomas, as well as other benign retrorectal tumours, are often completely asymptomatic [4,6,8,9]. They are usually found incidentally when performing a pelvic or rectal examination [4,9]. Retrorectal lipomas also might give nonspecific symptoms, mostly from the compression of pelvic structures, viscera and nerves. Symptoms depend on the size of the tumour, its localisation, extension [3]. Diagnosis and treatment of retrorectal tumours, including lipomas, remain difficult. Currently, computed tomography scan used in conjunction with magnetic resonance imaging scan is the gold standard in diagnosing retrorectal tumours [4]. These methods are used to find out the size, structure of the tumour, its relationship with surrounding organs that is a necessary information for planning surgical approach [3,4,8]. Biopsy should not be performed before a surgery [5,6]. All retrorectal tumours, including lipomas, should be resected [3,6,7]. In the presenting case laparoscopic surgery was indicated due to exclude malignant process, because it is difficult to differentiate lipoma from low-grade liposarcoma on magnetic resonance imaging [10]. There are several common approaches for resection of the retrorectal tumours: anterior approach, posterior approach and combined approach. The access and approach of the tumour depends on its size, location, structure, involvement of adjacent structures [4,6,9,11]. Surgery can be undertaken either by open or laparoscopic approach [11]. Laparoscopic approach is a safe alternative for benign tumours offering not only less post-operative pain and discomfort, shorter hospital stay, smaller incisions and less noticeable scars, but also excellent visualization of pelvic structures, safety and completeness of the resection [9]. Our case has been reported in line with the SCARE criteria [12].

## 2 Case report

A 42-year-old female patient (BMI – 23, non - smoker) was presented to our clinic with no severe complaints, for extirpation of retrorectal mass. 3 months ago, during the preventive gynecological examination, ultrasound analysis showed a mass incidentally found in the left side of the pelvis. Pelvic computed tomography with contrast was decided to perform. It revealed a well circumscribed adipose tissue mass with septums, which was located behind the uterus, at the left ovary projection and covers the entire pelvic cavity. The size of a mass was 11.5 cm × 6.5 cm in diameter. It presupposed diagnosis of dermoid cyst or lipoma. What is more, a congenital renal tract abnormality-duplicated collecting system, was detected. To clarify diagnosis and localization of lipoma the magnetic resonance imaging (MRI) was performed. It showed a giant retrorectal homogeneous adipose tissue opacity mass, surrounded by thin fibrous capsule, 12 cm × 6.7 cm × 8.6 cm in diameter, spreading toward the left obturator foramen (Photos 1). Surgery was indicated due to exclude malignant process certainly, because it is difficult to differentiate lipoma from lowgrade liposarcoma on MRI [2]. A laparoscopic extirpation of the retrorectal tumour was planned. We decided to perform laparoscopic approach instead of laparotomy to reduce postoperative complications, length of hospital stay and pain, furthermore, minimally invasive surgery brings better cosmetic results.

Informed patient consent had been obtained before the procedure. The patient was brought under general anesthesia with endotracheal intubation; surgery was performed by the laparoscopic approach. The patient was placed in the Trendelenburg position. After preparation of the surgical field and pneumoperitoneum formation, the camera port for video laparoscope (10 mm) was placed. Organs of the abdominal cavity were explored, some adhesions in the true pelvis and right iliac region were found. Under the control of the laparoscope, three trocars were introduced: one 12 mm trocar in the right iliac region and two 10 mm trocars on right and left sides of the paraumbilical region, 5 cm below the umbilicus. The harmonic scalpel and two bowel forceps were inserted. Rectum dislocated to the right side of pelvis and two ureters were found. The soft tumor arranged between rectum and the left wall of the bony pelvis was palpated. After the pelvic peritoneum was revealed and the anterior surface of the lipoma was exposed, the tumor was dissected from the mesorectum, presacral fascia and the lateral side of the bony pelvis (Photo 2, 3). The resected tumor was removed in a retrieval bag through the 12 mm in the right iliac region, which was enlarged for its delivery. One drain in the tumor bed was inserted. The trocar wounds were sutured. The tumor of the adipose tissue structure was approximately 15 cm × 10 cm × 8 cm (Photos 4). The overall operative time was 80 min. Histological examination of the removed tissue was performed and the final diagnosis of the pathology was lipoma. The operation and early postoperative period showed no major complications. The drain was removed on the 1st postoperative day. On the 2nd postoperative day, in a satisfactory condition, the patient was discharged from the hospital. Currently, two weeks after the surgery, there is no complications related to the surgery and the patient is feeling well.

## 3 Discussion

Retrorectal tumours in adults are rare, explaining the small number of reports in the literature. The true prevalence of retrorectal tumours in the general population is unknown because many of them are asymptomatic [[1], [2], [3], [4], [5], [6], [7]]. However, Jao et al. found that retrorectal tumours accounted for 1 in 40,000 hospital admissions [5]. Retrorectal lipoma is a relatively uncommon benign retrorectal tumour. Non classified, miscellaneous tumours account for 10–25% of all retrorectal tumors. Presacral lipomas are included in this group [4,7,8]. These tumours are more common in women and usually affect patients between 40 and 60 years of age [9].

Retrorectal tumours were reported for the first time in the middle of the 19th century and the first surgical resection of the tumour was made by Dr. Middledorpf [11]. These tumours derives from the different tissues that comprise the retrorectal space [3]. Lipoma is composed of mature adipose tissue [13]. The accurate etiology of lipomas is generally unknown. Presacral lipomas, as well as other benign retrorectal tumours, are often completely asymtomatic, therefore they may frequently be clinically unrecognised and diagnosed tardy [4,6,9,10]. They are usually found incidentally when performing a pelvic or rectal examination [4,9]. Many cases among the women are detected during preventive gynaecological examinations, as it was in our case as well [8]. Retrorectal lipomas also might cause nonspecific symptoms, mostly from the compression of pelvic structures, viscera and nerves. Symptoms depend on the size of the tumour, its localization and extension [3,4]. Patients may have a wide spectrum of complaints, such as rectal fullness, change in bowel habit, painful defecation, dysuria, faecal or urinary incontinence, sexual dysfunction, neurological symptoms in the lower back and perineum, pain. Rarely, retrorectal tumors may lead to obstructive labor and predispose the patient to life-threatening dystocia [4,6,7,10].

Diagnosis and treatment of retrorectal tumours, including lipomas, remain difficult. Rectal examination might give some suspicions of the likely diagnosis. According to the different studies [3,7,9] the majority of patients (75–100%) have a palpable retrorectal mass on digital examination. Computed tomography scan or magnetic resonance imaging should be performed in order to confirm the diagnosis. These methods are used to find out the size, structure of the tumour, its relationship with surrounding organs. It is a necessary information for planning surgical approach [3,4,9,14]. Currently, computed tomography scan used in conjunction with magnetic resonance imaging scan is the gold standard in diagnosing retrorectal tumours. Magnetic resonance imaging is not only essential in surgical planning but can assist in differentiation whether the tumour is likely malignant, benign or “uncertain” [4]. For this reason it avoids the need for routine preoperative biopsy [15].

The role of preoperative biopsy for retrorectal tumours is controversial [4,9]. Because of the reason that magnetic resonance imaging can identify lesions containing malignant transformation and the biopsy may cause infection, fecal fistula or increase the chance of tumour spread, there is a general rule that biopsy should not be performed [5,6]. If it is decided to approach the biopsy, a CT-guided extrarectal and presacral approach is recommended [9]. All retrorectal tumours, including lipomas, should be resected, even if the patient is asymptomatic and even though many tumours are benign [3,6,7]. The resection of the tumour confirms the diagnosis, eliminates the need for biopsy, prevent haemorrhage, infection, commpresion of the adjacent organs, chronic pain, dystocia during delivery and/or malignant transformation [3,6,16]. Lipomas, the benign retrorectal tumours, require complete gross resection [4].

There are several common approaches for resection of the retrorectal tumours: anterior approach (transabdominal), posterior approach (perineal) and combined abdominoperineal approach. The access and approach of the tumour depends on its size, location, structure, involvement of adjacent structures. Generally, tumours above the level of S3 or with invasion of adjacent structures will require an anterior or combined approach and *en bloc* resection; and lesions below the level of S3 will be resectable via the perineal posterior approach [4,6,10,14]. Surgery can be undertaken either by open or laparoscopic approach [14]. There is a little information on laparoscopic approaches to retrorectal tumours in adults [6]. Nevertheless, Konstantinidis et al. reported two cases of laparoscopic resection of presacral schwannomas and showed that laparoscopic approach is a safe alternative for benign tumours offering excellent visualization of pelvic structures [17]. Although there are no long-term studies that compare this method with other approaches [7]. The prognosis depends on the type of tumour and completeness of resection. The overall survival for benign retrorectal tumours is approximately 100% and if the resection is complete recurrences are rare [4,7,10]. Minimally invasive surgery gains more acceptances recently for resection of benign retrorectal lesions. Laparoscopy may be an alternative to the anterior abdominal approach and, in some cases, it can replace the combined approach. Literature reports a few specific advantages of minimally invasive surgery in retrorectal tumours resection in terms of safety, visualization of the retrorectal space and completeness of the resection. Nevertheless, there are laparoscopy advantages in general: it causes less post-operative pain and discomfort, shorter hospital stay, smaller incisions and smaller, less noticeable scars, less injury totissue [10].

To sum up, minimally invasive treatment of the large benign tumors of the pelvis is considered to be the method of choice. Despite the fact that the laparoscopic approach to large presacral tumours is challenging for the surgeon, the better visualization of the retrorectal space, less post-operative pain, shorter hospitalization time, less injury to tissue are the factors, which support the choice of this method. However, there are no long-term studies that compare this method with other approaches and more cases need to be gathered for further statistical analysis to prove the benefits of minimally invasive surgery in the treatment of retrorectal tumours.

## 4 Conclusions

In conclusion, a large, 15 cm in size, retrorectal lipoma can be successfully resected through the laparoscopic approach. We have found this minimally invasive operation to be an effective and well tolerated treatment option.

## Ethical approval

For case report not need reference number.

## Sources of funding

Nobody funding.

## Author contribution

Eligijus Poskus - study design, data collections, performed operation.

Gabija Makunaite – writing, data collections, data analysis.

Ieva Kubiliute – writing, data collections, data analysis.

Donatas Danys – writing, study design, data collections, data analysis, assisted operation.

## Conflicts of interest

No disclosure.

## Research registration number

None.

## Guarantor

Donatas Danys - accept full responsibility for the work, had access to the data, and controlled the decision to publish.

## Provenance and peer review

Not commissioned externally peer reviewed.

## Appendix A Supplementary data

SCARE Guideline Checklist SCARE Guideline Checklist
